# Fractured Pasts: The Structure of the Life Story in Sexual-Trauma Survivors With Posttraumatic Stress Disorder

**DOI:** 10.1177/2167702620917984

**Published:** 2020-06-18

**Authors:** Georgina Clifford, Caitlin Hitchcock, Tim Dalgleish

**Affiliations:** 1 Medical Research Council Cognition and Brain Sciences Unit, University of Cambridge; 2 Cambridgeshire and Peterborough National Health Service Foundation Trust, Cambridge, England

**Keywords:** autobiographical memory, cognitive processes, emotion, posttraumatic stress disorder

## Abstract

We examined the organization of past and future affective autobiographical knowledge in sexual-trauma survivors compared with control participants. Participants (*N* = 113) divided their past (and future) life into chapters (e.g., “college,” “marriage”), then characterized each chapter using positive or negative attributes. Sexual-trauma survivors (*n* = 27) endorsed a greater proportion of negative attributes, demonstrated greater affective compartmentalization (separation of positive and negative attributes into different chapters), and showed reduced redundancy (consistent endorsement across chapters) of positive attributes relative to control participants (*n* = 23). Groups did not differ on negative redundancy for past life structure or any metrics for future life structure. In a secondary analysis, we compared life structures for the sexual-trauma group and for individuals with chronic depression but no sexual-trauma history (*n* = 30) and matched control participants (*n* = 56), which revealed significantly greater negative redundancy in the depressed group. The distinct life structure presented by sexual-trauma survivors may reflect efforts to constrain the impact of trauma on an individual’s self-identity.

The experience of severe or repeated trauma has a fundamental impact on how the autobiographical past is remembered. A key distortion of autobiographical memory associated with trauma exposure is the experience of intrusive memories of the traumatic experience itself. These intrusions are prototypically high in frequency, fragmented, sensory laden, involuntary, distressing, and relatively immune to attempts to prevent them. Intrusions often take the form of images or thoughts but can also occur as “flashbacks”—the intense reliving of the original experience as if in the present moment. Such intrusions represent a cardinal symptom of posttraumatic stress disorder (PTSD; American Psychiatric Association, 2013; Brewin & Burgess, 2014), which is a common mental-ill-health response to severe or repeated trauma.

Trauma survivors therefore commonly exhibit a variety of different psychological and behavioral strategies to protect themselves against potentially toxic or damaging information stemming from their traumatic experiences and the impact on the self. Chronic use of these strategies is thought to be associated with a prolonged stress response following trauma given that many are included among the fifth edition of the *Diagnostic and Statistical Manual of Mental Disorders* (*DSM–5*; American Psychiatric Association, 2013) Avoidance Symptoms of PTSD. This includes avoiding reminders of the trauma, attempts not to think or talk about the trauma, social withdrawal, emotional numbing, loss of interest in particular activities, and psychogenic amnesia (American Psychiatric Association, 2013). Other strategies employed by trauma survivors to inhibit traumatic recollections and the overwhelming emotions associated with the trauma (Holmes et al., 2005) include dissociation (e.g., depersonalization and derealization), suppression, and repression.

However, these avoidance strategies may lead to a second key distortion of autobiographical memory in trauma survivors—a relative difficulty in accessing specific memories of all past events, whether positive, negative, or neutral in valence (e.g., Harvey, Bryant, & Dang, 1998; McNally, Lasko, Macklin, & Pitman, 1995; McNally, Litz, Prassas, Shin, & Weathers, 1994; for a review, see Moore & Zoellner, 2007). For example, in laboratory studies, when explicitly asked to retrieve a specific memory from their lives in response to a cue word (e.g., *happy*) on the Autobiographical Memory Test—the standard paradigm for examining memory specificity—participants with trauma histories are more likely to generate an overly general response (e.g., “Whenever I visit my friend”) instead of a memory of a single, circumscribed event (e.g., “Going to my friend’s place last Saturday afternoon”). The leading hypothesis is that this tendency for overgeneralized recollection of the past reflects an avoidant processing style that is initially focused on preventing detailed recollection of specific trauma memories but that has subsequently generalized to all autobiographical material (Williams et al., 2007). There is some support for this view; PTSD sufferers who exhibit higher levels of avoidance symptoms show the greatest overgeneralization when remembering nontrauma material (e.g., Kuyken & Brewin, 1995; Williams et al., 2007; Williams, Stiles, & Shapiro, 1999).

Finally, the autobiographical life story also needs to be updated after trauma to integrate the traumatic experience into the individual’s life narrative. For all of us, memories of past traumatic experiences are thought to provide meaning and structure to our life narratives as well as to help stabilize our conceptions of ourselves (e.g., Baerger & McAdams, 1999; Pillemer, 1998, 2003). However, if the trauma memory is not well integrated, it may form a central reference point for the individual’s whole life and identity and infuse other, nontraumatic experiences with trauma-related meaning (Bernsten & Rubin, 2006, 2007). Such meanings include negative beliefs about the self being broken or damaged by past traumatic experiences or the world being untrustworthy and toxic. In turn, these negative beliefs are a strong predictor of the development of PTSD following trauma (Halligan, Michael, Clark, & Ehlers, 2003).

The aim of the present study was to examine the structure of the autobiographical life story in survivors of repeated sexual trauma who have current, active, mental-health consequences (operationalized here as meeting criteria for a current diagnosis of PTSD) to further elucidate the impact of trauma on how individuals represent and organize information about their past. Life-story narratives are disrupted in a range of mental-health problems, and distortions have been observed in the affective content of narratives in borderline personality disorder, schizophrenia, and depression (e.g., Adler, Chin, Kolisetty, & Oltmanns, 2012; Alle et al., 2016; Dalgleish & Werner-Seidler, 2014). However, less is known about the structural organization of the autobiographical past after severe trauma despite the seminal models that guide treatment of PTSD ascribing an integral role to autobiographical memory in the development and maintenance of the maladaptive appraisals and beliefs that drive the disorder (Brewin, Dalgleish, & Joseph, 1996; Ehlers & Clark, 2000). Thus, greater understanding of how the overall structure of autobiographical memory is affected by severe trauma will have implications for theories of autobiographical memory (e.g., by indicating whether the strategies applied to trauma memories affect how individuals remember positive information about themselves) but also for treatment of trauma-related mental ill health such as PTSD. Findings will also indicate whether the autobiographical memory-based interventions that have shown promising effects in treating depression and anxiety (Hitchcock, Werner-Seidler, Blackwell, & Dalgleish, 2017) may also be useful for trauma survivors.

To examine the structure of the autobiographical life story, we used a card-sorting method originally formulated within the self-organization literature (Dozois & Dobson, 2001a, 2001b; Linville, 1985, 1987; Showers, 1992; Showers & Kling, 1996; Zajonc, 1960) but subsequently applied to study of the autobiographical past (Dalgleish, Hill, Golden, Morant, & Dunn, 2011; Jobson et al., 2018). The advantage of this approach is that rather than extracting metabeliefs about the life narrative using self-report, the task instead requires participants to map out their life structure and to think independently about each “chapter” of their lives (Conway, 2005; Thomsen & Berntsen, 2008). This allows us to examine patterns that manifest across the different chapters and that systematically differ between participants with a history of significant trauma relative to control participants without such a history.

## The Life-Structure Task

The central premise of the life-structure task (Dalgleish et al., 2011) is to ask participants to generate a list of the important time periods—life chapters—from their past (e.g., “my time at college,” “my marriage”). Participants are then asked to allocate sets of negative and positive adjectives (preselected as prototypical descriptors of life periods) to those chapters for which they are relevant. Life chapters have been identified as a component in theories of autobiographical memory (e.g., Conway & Pleydell-Pearce, 2000) that provide the basic scaffolding for mental representation of an individual’s life story. Life chapters are thought to contain knowledge and information relating to places, activities, and people associated with a particular period of time in an individual’s life and to be associated with a certain emotional valence (Conway, 2005; Conway & Pleydell-Pearce, 2000). Chapters appear to be of no fixed length; most last from months to years (e.g., Thomsen & Berntsen, 2008).

The life-structure task permits the computation of several metrics from which one might predict systematic differences for those whose personal narratives had been chronically shaped by the experience of trauma. The first is the overall relative proportions of negative and positive descriptors seen as applicable across chapters. Related to this are indices of positive and negative “redundancy”—the degree to which the same descriptors are repeatedly applied across all chapters (Linville, 1987). Finally, we can compute a measure of “compartmentalization”—the extent to which positive and negative descriptors are disaggregated into different life chapters. In the original study employing the life-structure task, Dalgleish et al. (2011) used these metrics and found that individuals with chronic and recurrent major depressive disorder (MDD) used a greater proportion of negative cards and showed greater negative redundancy, reduced positive redundancy, and greater compartmentalization in the way the cards were allocated relative to a never-depressed control group across their life structure.

In the present study, we selected participants with a chronic history of sexual violence. We focused on sexual assault or abuse because we anticipated that the long-lasting effects of such significant interpersonal trauma might have the clearest effects on overall life structure (Herman, 1992). We compared this group with control participants who had no such history of sexual assault or abuse trauma and no history of posttraumatic stress.

We predicted that our participants with a history of sexual trauma would use a greater proportion of negative descriptors across their life structures. Given the extensive literature describing avoidant and dissociative psychological strategies to manage negative information about the past in trauma survivors, we also anticipated that there would be greater compartmentalization of positive and negative information in sexual-trauma-exposed participants relative to our control group. Our hypotheses concerning the redundancy metrics were less clear. There is evidence of reduced life satisfaction and well-being in people exposed to trauma (e.g., Karatzias et al., 2013; Richardson, Long, Pedlar, & Elhai, 2008), but it is unclear whether this would translate to impoverished positive themes across the life structure. Likewise, although chronic PTSD is characterized by the endorsement of dysfunctional higher-order meanings pertaining to the trauma (e.g., “My life has been destroyed”; Foa, Ehlers, Clark, Tolin, & Orsillo, 1999; for a discussion, see Park, 2010) and evidence of increased centrality of traumatic events in the life narrative (Bernsten & Rubin, 2006, 2007), it is again unclear to what extent severe trauma would affect the development of negative themes more generally across the life structure. We therefore had no strong hypotheses concerning the redundancy metrics.

To evaluate the putative specificity of the profile of performance across the life-structure metrics associated with sexual trauma, we also planned to compare the current sexual-trauma-exposed sample and control participants with a chronically depressed sample with MDD and associated control participants pooled from prior studies (Dalgleish et al., 2011; Werner-Seidler et al., 2018) given that the method and research setting were the same.

## Future Life Structure

In a study using the life-structure task, Dalgleish et al. (2011) also asked depressed and control participants to generate anticipated future chapters of their life and allocate the same positive and negative descriptors to these future periods. Dalgleish et al. found no significant differences between the MDD and control groups regarding the organization of putative future life chapters. Thus, we also examined future life-structure metrics in our participants with and without a sexual-trauma history, but we had no clear hypotheses given the previous data.

## Method

### Participants

We based our power calculations for minimal sample-size estimations per group on the smallest effect size (Cohen’s *d* = 0.96) for the past-life-structure metrics between the MDD and control groups in Dalgleish et al. (2011). With α set at .05 and 80% power, two-tailed, the power calculation indicated sample sizes of 19 per group.

Our initial analyses compared 27 women with a history of sexual assault or abuse (the trauma group) with a group of 23 control participants with no such trauma history *and* no current or lifetime history of PTSD (the control group). Presence of sexual trauma, PTSD diagnosis, and other Axis I and II psychiatric comorbidity were determined according to the fourth edition of the *Diagnostic and Statistical Manual of Mental Disorders* (*DSM–IV*; American Psychiatric Association, 1994) using the Structured Clinical Interview for the *DSM–IV* Axis I Disorders–Clinician Version (SCID, Version 2.0; First, Spitzer, Gibbon, & Williams, 1996) and the Structured Clinical Interview for *DSM–IV-TR* Axis II Personality Disorders (Borderline, Avoidant, and Dependent; First et al., 1997). Interviews were administered either by trained research staff under the supervision of a clinical psychologist or by a clinical psychologist.

Nineteen participants were recruited from The Haven: A Sexual Assault Referral Centre in London, United Kingdom. They were invited to take part after attendance at The Haven follow-up clinic or during an assessment for counseling or psychological therapy. The remaining 8 participants were recruited from the Cognition and Brain Sciences Unit, Cambridge, Volunteer Mental Health Panel—a database of some 400 community volunteers with a history of mental health problems who have agreed to help with psychological research. Volunteers are recruited to the panel via advertisements online and in local newspapers.

As is typical for clinical groups with a history of sexual trauma, in addition to all 27 women having a PTSD diagnosis, there was notable additional psychiatric comorbidity in the trauma group. According to the SCID, in the trauma group, 7 participants also met criteria for a current episode of MDD, 25 met the criteria for a past episode of MDD, 8 met the criteria for current panic disorder (secondary to PTSD), 3 met the criteria for current agoraphobia, 5 met the criteria for current borderline personality disorder, and 3 met the criteria for current avoidant personality disorder.

The control group participants (*n* = 23) had no self-reported history of sexual trauma and no history of PTSD according to the SCID, although 3 participants met the criteria for a past major depressive episode and 1 for current panic disorder. The control participants were recruited from the Cognition and Brain Sciences Unit, Cambridge, Volunteer Panel—a database of some 2,000 community volunteers who have agreed to help with psychological research. Volunteers are recruited to the panel via advertisements online and in local newspapers.

To be eligible for the study, participants had to be fluent in English and over 18 years old. Exclusion criteria comprised a current diagnosis of substance dependence, a history of psychosis, or organic brain injury, all assessed via the SCID. No participants were excluded on these bases.

### Materials and measures

#### Life-structure task

The life-structure task was delivered as used by Dalgleish et al. (2011). Participants were first asked to imagine that they had to write their autobiography and in preparation they should divide their past life into chapters. Participants were told that they were free to create as many chapters as they felt were appropriate, that chapters did not need to have a clear beginning and end, and that chapters could run in parallel with other chapters. They were also informed that ongoing life chapters could be included. Participants were given a blank table and asked to record their life chapters at the top of each column and to include their age at the beginning and end of each chapter.

Participants were then given a deck of 48 cards, each containing an adjective or phrase that might be used to describe a period of one’s life. Some of these adjectives differed slightly from Dalgleish et al.’s (2011) study to allow us to reference affective states that trauma survivors endorse, for example, “feeling contaminated,” “feeling broken,” and “feeling dirty.” The adjectives/phrases were either positive or negative in valence (24 of each; see Appendix A). Before the study, we had the adjectives and phrases rated (*N* = 15 unselected raters) for valence on 15-point Likert scales (1 = *strongly positive*, 7 = *weakly positive*, 8 = *neutral*, 9 = *weakly negative*, 15 = *strongly negative*). The positive set of adjectives had a mean rating of 2.59 (*SD* = 0.81), whereas the negative set of adjectives had a mean rating of 13.61 (*SD* = 1.09). An independent-samples *t* test showed that the two sets of cards did not differ significantly in intensity (distance from the neutral score of 8; *t* < 1).

For the card sort, participants were asked to allocate cards (adjectives and phrases) that they felt were relevant to each of the life chapters identified (for further details on the card-sorting procedure, see Dalgleish et al., 2011).

Participants were next asked to imagine their future life structure—the chapters of their life that were potentially still to come (e.g., “retirement,” “grandchildren”) and to repeat the card-sorting procedure for the future life chapters.

#### Life-structure-task metrics

##### Proportion of negative cards

This measure is the number of negative attributes, including repetitions, appearing in the card sort divided by the total number of attributes used. It is a measure of the overall negativity of the card sort (Showers, 1992).

##### Compartmentalization

Compartmentalization (Showers, 1993) is calculated as a φ coefficient on the basis of a χ^2^ statistic (Everitt, 1977). It compares the frequencies of negative and positive cards in each chapter with those that would be expected given the proportion of negative items for the card sort as a whole. A frequency table is constructed that contains as many columns as there are chapters in the individual’s card sort and two rows for number of positive cards and number of negative cards. The observed frequencies for each cell are generated from the whole card sort. The expected frequencies are generated as follows: If the card sort contained, for example, 40% negative cards overall and the first chapter contained 20 cards, then the expected frequencies for that chapter would be 8 (40%) negative cards and 12 (60%) positive cards. A χ^2^ statistic is then computed using these expected and observed frequencies. This is then normalized by dividing by the number of cards in the sort (*N*) as follows:

$$\Phi \;=\;\frac{\sqrt{\chi^{2}}}{N},$$

where φ can range from 0 to 1 (0 represents a perfectly random sort, and 1 represents a perfectly compartmentalized sort).

##### Redundancy

Redundancy (Dozois & Dobson, 2001a, 2001b) was computed separately for positive and negative attributes; each redundancy score represented the degree of card repetitions across chapters controlling for both the number of chapters in a given card sort and the number of cards used. The following formula generated the redundancy rates:

$$\mathrm{Redundancy}\;=\;x\;=\;\frac{1}{n_{\mathrm{dw}}\;\times \;n_{\mathrm{dg}}}\;\times \;\sum n_{\mathrm{ri}},$$

where (using the example of negative redundancy) *n*_dw_ equals the number of distinct negative words used in an individual’s card sort, *n*_dg_ equals the number of chapters generated, and *n*_ri_ equals the sum of repetitions of each negative card up to the maximum of 23 cards.

### Procedure

Ethics approval was obtained from the NHS National Research Ethics Service (reference 11/H0305/1). Participants completed the tasks and measures individually and face to face with the experimenter in a quiet testing room. After provision of informed consent, participants completed the SCID, a semistructured interview on auditory pseudohallucinations (not reported here), and several self-report questionnaire measures of mood and PTSD symptoms. In a separate session, approximately a week later, they completed the life-structure task.

### Screening and questionnaire measures

The following measures provided an overview of emotional disturbance.

*The Complex Trauma Symptoms Questionnaire* (CTSQ; Cloitre et al., 2014) is a 49-item measure that indexes symptoms of complex PTSD. The measure has been previously used to index symptoms of complex PTSD in women with a history of interpersonal violence (Cloitre et al., 2014). Internal consistency was high in the current sample (α = .97).

The Beck Depression Inventory (BDI; Beck, Ward, Mendelson, Mock, & Erbaugh, 1961) is a widely used and well-validated measure of depressive symptoms over the previous week.^1^ The BDI demonstrates high internal consistency; α coefficients were .86 and .81 for psychiatric and nonpsychiatric populations, respectively (Beck et al., 1988). Internal consistency was high in the current sample (α = .96).

The Centrality of Events Scale (CES-negative; Berntsen & Rubin, 2006, 2007) measures the extent to which a traumatic memory forms a central component of personal identity, a turning point in the life story, and a reference point for everyday inferences. We used the full version, which consists of 20 items rated on 5-point scales (1 = *totally disagree*, 5 = *totally agree*) in relation to the most stressful or traumatic event in the person’s life. The CES-negative is positively correlated with severity of PTSD symptoms, which remain significant when controlling for measures of anxiety, depression, dissociation, and self-consciousness (Berntsen & Rubin, 2006, 2007). Internal consistency was high in the current sample (α = .98).

## Results

### Statistical analysis

Our coprimary analyses were two multivariate analyses of variance (MANOVAs) of between-group differences in life structure metrics for (a) past chapters and (b) future chapters, respectively. Use of multiple analyses of variance (MANOVA) allowed us to simultaneously evaluate differences in compartmentalization and redundancy for both positive and negative attributes. Follow-up univariate analyses of variance (ANOVA) were planned contingent on the statistical significance of the initial MANOVAs to determine on which of these metrics the trauma and control groups significantly differed.

Our secondary analyses repeated these MANOVAs to assess between-group differences between our trauma group, the MDD group collected by Dalgleish et al. (2011), and control participants who had a history of neither depression nor sexual trauma.

### Descriptive data

Descriptive group data are presented in Table 1. The groups did not differ in age, *t*(48) = 0.64, *p* = .52, *d* = 0.18, 95% confidence interval (CI) = [–0.40, –0.76], but did differ significantly in education level, *t*(47.53) = 2.89, *p* = .006, *d* = 0.82, 95% CI = [0.22, 1.42]. As expected, there were differences in BDI scores, *t*(27.16) = 9.19, *p* < .001, *d* = 2.61, 95% CI = [1.82, 3.40], and CES scores, *t*(43.61) = 9.83, *p* < .001, *d* = 2.79, 95% CI = [1.97, 3.61], in the trauma and control groups. Because our trauma and control samples were not matched on education level, we repeated all analyses with education levels covaried. Results remained the same. We therefore present the analyses without the covariate.

**Table 1.** Descriptive Data for the PTSD Group and No-PTSD Control Group

Statistic	PTSD group (*n* = 27)	No-PTSD control group (*n* = 23)
Years of education	14.15 (2.87)	16.48* (2.27)
Age (in years)	37.63 (13.17)	35.09 (14.87)
Complex Trauma Symptoms Questionnaire	105.93 (48.93)	4.74* (5.00)
Beck Depression Inventory	26.07 (12.70)	1.52* (1.76)
Centrality of Event Scale	82.19 (15.27)	36.13* (17.87)

Note: Values are means with standard deviations in parentheses. PTSD = posttraumatic stress disorder.

* *p* < .05 for between-groups difference.

### Primary analyses

#### Past life structure in sexual-trauma survivors

All participants were able to generate multiple chapters to describe their past lives (minimum = 3). Examples of chapter titles were “learning first steps,” “school years,” “marriage,” “the grand challenge,” “a new beginning,” and “what it all means.”

Data concerning numbers of chapters generated and numbers of cards used are presented in Table 2. The groups did not significantly differ in the number of past chapters they generated, *t*(48) = 0.57, *p* = .57, *d* = 0.16, 95% CI = [–0.42, 0.74], or in the total number of cards used in the past card sort, *t*(48) = 1.13, *p* = .26, *d* = 0.32, 95% CI = [–0.26, 0.90]. This suggests broadly comparable engagement in the task across groups and indicates that any group differences on the structure metrics considered below were not a simple function of numbers of chapters or cards employed.

**Table 2.** Number of Past and Future Chapters and Cards Used in the Past and Future Card Sorts by Group

Measure	PTSD group (*n* = 27)	No-PTSD control group (*n* = 23)
Past chapters (range = 3–16)	8.74 (3.18)	9.26 (3.22)
Cards used in past sort (range = 11–266)	81.67 (51.95)	98.70 (54.42)
Future chapters (range = 1–9)	4.26 (2.30)	5.04 (2.44)
Cards used in future sort (range = 2–173)	40.30 (30.19)	57.39 (41.25)

Note: Values are means with standard deviations in parentheses. All between-group differences are nonsignificant. PTSD = posttraumatic stress disorder.

**Fig. 1.**
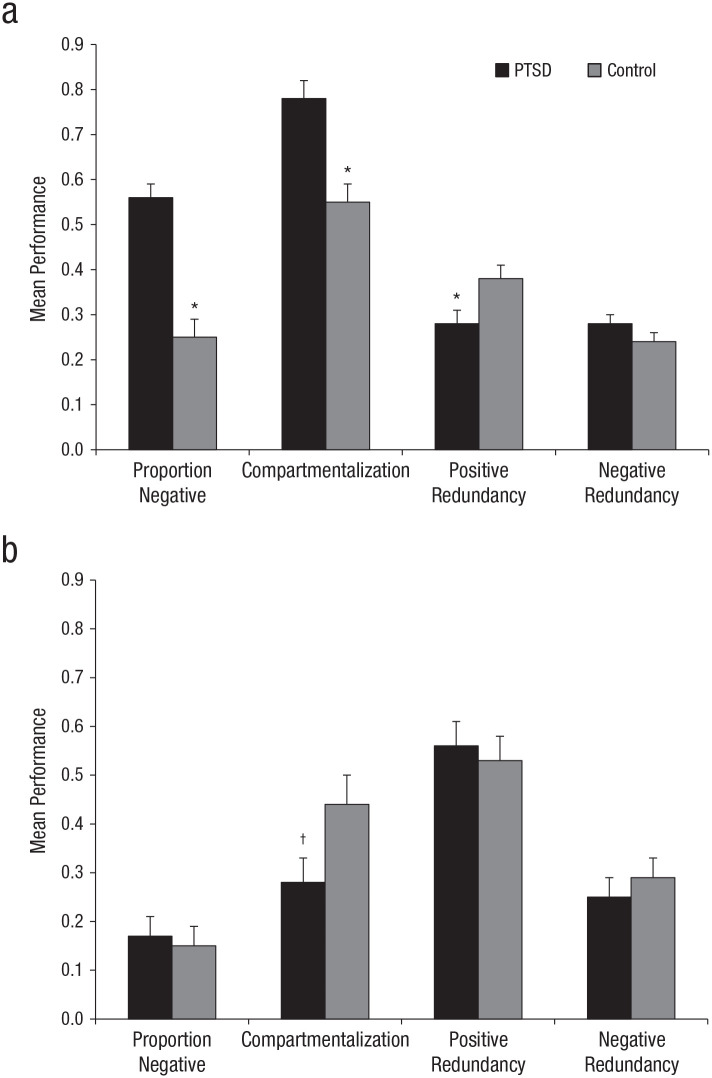
Mean performance for proportion of negative cards used, positive and negative redundancy, and compartmentalization for the past-life chapters (a) and for the future-life chapters (b), separately for the posttraumatic-stress-disorder (PTSD) group and no-PTSD control group. Error bars represent standard errors. Symbols represent significant differences between the PTSD and control groups (*p < .05) and an effect that became nonsignificant once it was covaried for education (^†^*p* > .05).

The past-life-structure metrics for the trauma and control groups are presented in Figure 1a. There were broad ranges of scores across both groups on the four past-life-structure metrics (maximum possible range = 0–1), which suggests that across-group floor and ceiling effects were not evident; proportion of negative cards = 0.02 to 0.93, negative redundancy = 0.12 to 0.45, positive redundancy = 0.09 to 0.72, and compartmentalization = 0.22 to 1. To illustrate the raw data, Appendix B shows two examples of actual past card sorts demonstrating lower and higher levels of compartmentalization. Of particular note, in the more integrated card sort example (low compartmentalization) in Appendix B1, several of the life chapters contain positive and negative descriptors that are diametrically opposite in meaning.

To test our hypotheses and assess whether past life structure differed across the two groups, we first conducted a MANOVA (see Dalgleish et al., 2011) across groups with the four past-life-structure metrics as the dependent variables. There was a statistically significant multivariate difference for the past-life-structure components across groups, Wilks’s Λ = .49, *F*(4, 45) = 11.80, *p* < .001, *d* = 0.97, 95% CI = [0.36, 1.58].

Follow-up univariate ANOVAs indicated a significantly greater proportion of negative cards were used, *F*(1, 48) = 39.19, *p* < .001, *d* = 1.78, 95% CI = [1.09, 2.47]; significantly greater compartmentalization, *F*(1, 48) = 18.88, *p* < .001, *d* = 1.23, 95% CI = [0.60, 1.86]; and significantly reduced positive redundancy, *F*(1, 48) = 7.59, *p* = .02, *d* = 0.78, 95% CI = [0.18, 1.38], for the trauma group relative to the control group. There was no significant difference between groups on negative redundancy, *F*(1, 48) = 2.18, *p* = .15, *d* = 0.42, 95% CI = [–0.17, 1.01].

#### Future life structure in sexual-trauma survivors

We next examined performance on the future-life-structure task for the trauma and control groups. All participants generated multiple future life chapters. Some examples of chapter headings were “hard times,” “new career,” “grannies and chickens,” “children married,” “parent death,” “death of spouse,” “holidays abroad,” and “grandchildren.” The future-life-structure data are presented in Table 2 and Figure 1b. The across-group ranges of scores on the future-life-structure metrics were broad, similar to the past metrics. Proportion of negative cards ranged from 0 to 0.94; negative redundancy = 0 to 1, positive redundancy = 0.18 to 1, and compartmentalization = 0 to 1. In addition, in line with the past metrics, the groups were not significantly different on the number of future chapters, *t*(48) = 1.17, *p* = .25, *d* = 0.33, 95% CI = [–0.25, 0.91], or on total number of cards used in the future sort, *t*(48) = 1.69, *p* = .10, *d* = 0.48; 95% CI = [–0.11, 1.07]. A MANOVA on the future metrics revealed no statistically significant differences in the future-life-structure components between groups, Wilks’s Λ = .89, *F*(4, 45) = 1.45, *p* = .23, *d* = 0.34, 95% CI = [–0.24, 0.92].^2^

#### Correlations between PTSD symptoms and past-life-structure metrics

To begin to explore whether these disruptions to life structure were related to PTSD symptom severity rather than simply trauma exposure (given that our trauma and control groups differed on both variables), we next analyzed correlations between symptom severity on the CTSQ and each of the metrics (reduced positive redundancy, increased proportion of negative cards, increased compartmentalization; Fig. 1a) that significantly differed across the two groups. Within the trauma group, we found significant correlations between increased PTSD symptoms and both higher proportion of negative cards, *r*(25) = .50, *p* = .008, and lower positive redundancy, *r*(25) = −.41, *p* = .04, but no support for a correlation between symptom severity and compartmentalization, *r*(25) = −.09, *p* = .64. As expected, all of these relationships were nonsignificant in the control group, in which the CTSQ was completed with respect to the self-identified most significant past negative event, *p*s > .4.

### Secondary analyses

#### Comparing

individuals with a history of sexual trauma and a primary diagnosis of chronic MDD relative to control participants for past life structure.

It is important to determine whether the observed pattern of results is unique to the experience of sexual trauma or associated with psychopathology more broadly. The present findings exploring past life structure in individuals with chronic sexual-trauma histories show a different profile to earlier research using the same task with individuals with a long-term diagnosis of chronic MDD (for details, see Dalgleish et al., 2011). Specifically, both groups show similar patterns relative to control participants in terms of proportion of negative cards used, compartmentalization of the card sort, and positive redundancy across life chapters. The MDD participants, however, also showed enhanced negative redundancy—the tendency to endorse the same trait constructs across multiple life chapters—relative to control participants. This effect was not observed in the current trauma group (see Fig. 1a).

Because the procedure for the MDD study (see Dalgleish et al., 2011) was almost identical to the current study and the two studies were conducted in the same research setting by the same research team, our secondary aim was to statistically compare the prior MDD and current trauma groups, against control participants, to further evaluate these apparent differences in life structure across the two clinical samples. That is, we sought to differentiate between the effect of severe sexual trauma (with associated PTSD) and non-PTSD psychopathology in the absence of sexual trauma. Our hypothesis was that performance on the negative-redundancy metric would differ significantly across the clinical groups, which would indicate that the tendency to assign greater negative descriptive regularity across the life structure was confined to recurrent depression. Specifically, we anticipated no differences between the two clinical groups on proportion of negative cards, compartmentalization, or positive redundancy in contrast to a predicted difference in negative redundancy. We did, however, expect the trauma group to differ from control participants on all life-structure metrics except negative redundancy, and we hypothesized that the MDD group would differ from control participants on all metrics.

To do this, we set aside those participants (*n* = 7) from the current trauma group who also met criteria for a diagnosis of current MDD to create a trauma/no-MDD group (*n* = 20). We also excluded data from the 3 participants in our current control group who met criteria for a past major depressive episode (American Psychiatric Association, 2013) to create a no-trauma/no-MDD control sample (*n* = 20). We next pooled participants with chronic MDD from two data sets in which we had used the past-life-structure task (Dalgleish et al., 2011; Werner-Seidler et al., 2018) but removed any participants with a self-reported sexual-trauma history or who met criteria for present or past PTSD to create a chronic, recurrent depression group (*n* = 30). Finally, we also removed participants from the MDD study control samples with current or past PTSD to create a no-MDD/no-PTSD control group (*n* = 36). We therefore analyzed data for four groups: trauma/no-MDD, depression, a control group from the current trauma-focused study, and a control group from the MDD study. We completed a series of validity checks of the original life-structure profiles for the trauma/no-MDD and depression groups and the two control groups (see the Supplemental Material available online). Note that there were no significant differences between the two control groups on any of the past-life-structure metrics, *F*s(1, 53) < 2.49, *p*s > .12, *ds* < 0.44. We therefore combined the two control groups into a single combined control group (*n* = 56) for our main analysis.

**Fig. 2.**
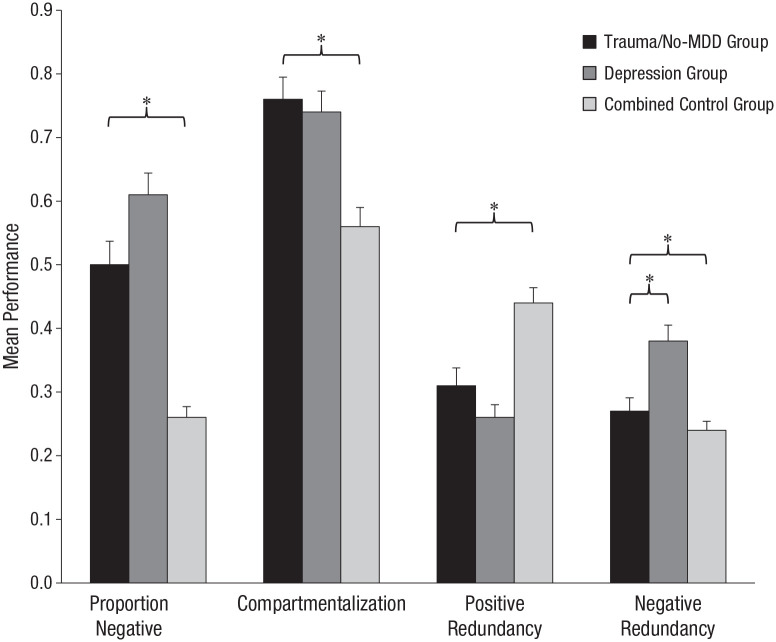
Mean performance for proportion of negative cards used, positive and negative redundancy, and compartmentalization for past-life chapters, separately for the trauma/no-MDD, depression, and combined control groups. PTSD = posttraumatic-stress-disorder; MDD = major depressive disorder. Error bars represent standard errors. Asterisks represent statistically significant differences between groups (*p < .05).

We proceeded with three groups for our key analyses, each with sample sizes that remained in line with our a priori power calculations: a sexual-trauma/no-MDD group (*n* = 20), a depression group (*n* = 30), and a combined control group (*n* = 56). The past life structure metrics for the three groups are presented in Figure 2. We conducted a multivariate analysis of covariance^2^ across the three groups with the four life-structure metrics as the dependent variables with age and gender covaried. There was a statistically significant difference for past-life-structure components across groups, Wilks’s Λ = .42, *F*(8, 196) = 13.25, *p* < .001. Follow-up univariate ANOVAs demonstrated significant effects of group for all four of the life-structure metrics, *F*s(2, 101) > 12.65, *p*s < .001.

Breaking down these effects revealed, as expected, that the depression group differed from the combined control group on the multivariate analysis, Wilks’s Λ = .45, *F*(4, 79) = 24.51, *p* < .001, *d* = 1.12, 95% CI = [0.63, 1.61], and on all four univariate metrics, *F*s(1, 82) > 14.52, *p*s < .001, *d*s > 0.80, which mirrors the previous MDD findings (Dalgleish et al., 2011). In addition, as expected, the trauma/no-MDD group differed from the combined control group on the multivariate analysis, Wilks’s Λ = .61, *F*(4, 69) = 10.95, *p* < .001, *d* = 0.86, 95% CI = [0.31, 1.41]. As with the initial trauma-sample analyses reported earlier, the univariate analyses demonstrated no significant differences between the trauma/no-MDD and combined control groups for negative redundancy, *F* < 1, but significant group differences for positive redundancy, proportion of negative cards, and compartmentalization, *F*s(1, 72) > 8.53, *p*s < .005, *d*s > 0.70.

Finally, the critical comparison between the two clinical groups—the trauma/no-MDD and depression groups—revealed a significant multivariate effect, Wilks’s Λ = .45, *F*(4, 43) = 2.85, *p* = .04, *d* = 0.49; 95% CI = [–0.11, 1.09]. The univariate analyses revealed that the two clinical groups did not differ significantly on either positive redundancy, *F*(1, 46) = 1.24, *p* = .27, *d* = 0.32, 95% CI = [–0.27, 0.91], or compartmentalization, *F* < 1. There was a nonsignificant but moderately sized effect for the depression group to select a higher proportion of negative cards, *F*(1, 46) = 3.66, *p* = .06, *d* = 0.55, 95% CI = [–0.05, 1.15]. Note that there was a significant difference indicative of greater negative redundancy in the depression group relative to the trauma/no-MDD group, *F*(1, 46) = 8.42 *p* < .01, *d* = 0.84, 95% CI = [0.22, 1.45]. Taken together, these findings indicate that the life-structure profiles of participants with chronic MDD and participants with a severe sexual-trauma history (with associated PTSD), although broadly similar, also critically differ in specific ways.

## Discussion

The primary aim of this study was to examine the organization of past autobiographical knowledge in a sample of sexual-trauma survivors compared with a sample of control participants with no history of sexual trauma or of PTSD by using a self-descriptive card-sorting task (Dalgleish et al., 2011). The secondary aim was to compare our findings in this sample with previously collected data on the same task from individuals with chronic, recurrent MDD.

In terms of our primary analyses, we found that participants with a history of sexual trauma (with associated PTSD) used a greater proportion of negative descriptors across their past life structure and showed greater compartmentalization of positive and negative information across chapters, which is consistent with our predictions. We had no strong directional a priori hypotheses concerning the past-life-structure redundancy metrics and found no significant differences for negative redundancy between the trauma and control groups but significantly greater positive redundancy for the control group relative to the trauma group. For the future card sort, we found no significant differences on any life-structure metrics between the trauma and control groups.

These primary findings for past life structure for sexual-trauma survivors, relative to control participants, were ostensibly different to earlier research using the same card-sorting task with individuals with a long-term diagnosis of MDD (Dalgleish et al., 2011). The key difference is that we found no support in our sexually traumatized sample for the enhanced negative redundancy that characterizes MDD participants relative to control participants. We therefore statistically compared the trauma survivors’ data against the prior MDD participant data, relative to control participants, and indeed found a significant difference indicative of greater negative redundancy in the MDD group relative to the trauma/no-MDD group and no significant differences in other aspects of the life structure. The trauma/no-MDD sample did contain some individuals with past experience of depression. However, any influence of past depression in this group (and indeed of past trauma in the MDD group) would have “gone against the grain” of our hypothesized clinical-group difference concerning negative redundancy. This gave us confidence that the presence of these clinical features would not confound the interpretation of the analytic findings given that such features should have served only to dilute any effects rather than spuriously drive them.

The current findings are notable in three important ways. First, they indicate that sexual-trauma survivors with PTSD structure their autobiographical narrative fundamentally differently to control participants with no exposure to sexual trauma. This is consistent with the notion that profound trauma and its sequelae markedly alter the sense of personal identity (e.g., Bernsten & Rubin, 2006, 2007). It is important to note that we were unable to conclusively differentiate the effects of sexual-trauma exposure per se and PTSD on narrative structure, although correlational analyses revealed that PTSD symptom severity did affect both use of negative cards and positive redundancy; greater symptoms were associated with higher scores on these metrics. Second, the narrative structure is not simply rendered more negative following the impact of sexual trauma but is also more compartmentalized; positive and negative life epochs are relatively more isolated from one another compared with the life structure in control participants. Correlational analyses revealed no support in the trauma group for an association between PTSD symptom severity and compartmentalization, however. Finally, there appears to be some specificity with respect to clinical presentation; the elevated negative redundancy that is characteristic of chronic depression did not emerge as a feature of the life structure in participants exposed to severe sexual trauma with resultant PTSD, which suggests that alterations in life structure are not simply a broad feature of mental health problems per se.

The greater overall negativity of the life structure after sexual-trauma exposure is perhaps the least surprising of the current results given that all such trauma-exposed participants were experiencing a chronic life-changing mental health problem (PTSD) rooted in profoundly traumatic experiences, the sequelae of which we know are characterized by pervasive negative affect and cognitions. As suggested in the introduction, the higher levels of affective compartmentalization observed in the sexual-trauma sample are consistent with aspects of the clinical presentation of PTSD after such trauma and may have generalized from an initial segregation of traumatic experiences as a way of “ring fencing” off traumatic information (Holmes et al., 2005) to a broader disaggregation of positive and negative evaluative information more generally. This is consistent with the data and theory in other cognitive domains such as overly general autobiographical memory (see Moore & Zoellner, 2007).

The reduced positive redundancy effect in the trauma group indicates that there are fewer consolidated positive themes running through the life narrative for participants with PTSD after sexual trauma relative to control participants. We have proposed that higher positive redundancy is reflective of augmented well-being and positive mental health, as opposed to merely the absence of negative mental health (Dalgleish et al., 2011; Dalgleish & Werner-Seidler, 2014). In this light, the lack of positive redundancy in the present findings concords with other evidence of reduced well-being in people with severe trauma histories (e.g., Karatzia et al., 2013; Richardson et al., 2008) and is perhaps unsurprising given the chronically disrupted lifestyles associated with the experience of sexual trauma.

Why did we find no support for a difference in negative redundancy between our sexual-trauma-exposed and control groups but a significant difference between our trauma sample and a chronic MDD sample with the latter showing elevated negative redundancy? This profile suggests that in our sample exposed to sexual trauma, distressing or toxic information relating to past negative experiences is more effectively segregated across the past life structure than for individuals with chronic MDD such that instead of pervading the individual’s entire history of personal experiences, the negative information is prevented from contaminating the other, more positive life epochs. This fits with the clinical presentation of PTSD after sexual trauma in which pervasive and chronic avoidance of trauma-related information and its consequences, via behavioral and lifestyle changes to more profound dissociative phenomena, can give rise to oases of healthy functioning (Dalgleish, 2004; Holmes et al., 2005). In contrast, for those with MDD, negative affect and information are characteristically less contained and pervade all aspects of the self, world, and future (Beck, Rush, Shaw, & Emery, 1979).

Note that although the individuals with sexual-trauma histories and associated PTSD showed clear differences from control participants in their narratives about the past, their perception of their personal future did not differ from that of control participants. This mimics previous findings in individuals with chronic depression (Dalgleish et al., 2011). It is unclear why the structural differences concerning the past found in sexual-trauma-exposed and depressed individuals do not extend to the future. The strong past-structure findings indicate that the absence of a future effect is unlikely to be an artifact of the general method used. One possibility is that prospective life-chapter predictions are populated in accordance with “cultural life scripts” (Berntsen & Rubin, 2004) that emphasize positivity (e.g., benefits of retirement, joys of grandchildren, positive events happening to offspring) and that these effects predominate even in the presence of generalized negative affect (e.g., hopelessness) about the future. This idea is consistent with work by Grysman and Dimakis (2018), who found that older participants, when composing scripts about their future life narrative, tended to generate a relatively restricted set of predominantly positive events—a finding the authors interpreted as a cultural life script operating in the service of future well-being despite anticipated physical and cognitive decline associated with later life. This projection of cultural scripts onto the individual life narrative is of course much more difficult for past events in which the reality of what has happened (e.g., profound trauma) will generally provide a more powerful organizing principle.

The present results indicate a clear empirical pathway to clinical translation. The first question to be addressed is the extent to which these changes in life structure drive and maintain PTSD symptoms over time within longitudinal cohorts of trauma survivors. If future studies suggest a causal role for autobiographical structure in driving symptoms, the next question is how effective it would be to work directly with the life-structure task. This may involve modifying generated life structures to integrate positive and negative material within each chapter to enhance positive redundancy across chapters with a view to ameliorating outcomes (Dalgleish & Werner-Seidler, 2014). Current “gold-standard” treatments for PTSD (e.g., trauma-focused cognitive behavioral therapy, narrative exposure therapy) have a strong autobiographical memory element given that they involve working with the trauma memory and more broadly contextualizing it within the life story. Our findings suggest that working directly with non-trauma-related (i.e., positive) elements of autobiographical memory and explicitly integrating them into the representations of discrete lifetime periods may also be useful for people experiencing PTSD after sexual trauma. Indeed, autobiographical memory-based interventions that are not trauma-specific have shown promising effects on PTSD symptoms (Hitchcock et al., 2017).

The study has some potential limitations. Although we did ask participants to describe their own life chapters, we did not ask them to produce their own descriptive words for the cards used in the sorting task. This decision was made because we wanted to ensure that there were comparable numbers of positive and negative cards to select from and also that the intensity of descriptors was comparable across participants so that we could draw conclusions about the life structure as opposed to the language used to describe it. Future studies could ask participants to provide their own adjectives to allocate to each of the cards used in the card sort that could then be rated in terms of valence and coded using metrics similar to those employed here (Rubin & Berntsen, 2003).

Our choice to work with participants with a chronic history of interpersonal trauma had implications for our selection of control participants because it is very difficult to find survivors of such experiences with no significant symptoms of past or current posttraumatic stress to act as a trauma-matched control sample. We therefore recruited control participants who had no such history of sexual assault or abuse trauma and no history of PTSD to any trauma. This means that it is difficult to disentangle whether it is the development of PTSD rather than the trauma history per se that accounts for our primary findings. We did explore this question using correlational analyses within the trauma group. Although the proportion of negative cards endorsed and the reduced positive redundancy across the life structure were significantly correlated with PTSD severity, this was not the case for the degree of compartmentalization, which suggests that PTSD severity is not the driving force behind the current profile of findings. Future studies could examine the replicability of the effects with survivors of more discrete or less severe trauma, which would also enable greater generalization of the effects from severe interpersonal trauma to other trauma categories.

A further limitation of the life-structure task is that although it focuses on the whole life narrative, it remains retrospective. The life-reconstruction approach with its mandate to generate individual chapters and consider them separately is an advance over less structured methods, but the possibility remains that contemporaneous consideration of past life chapters may have generated a different profile of findings. However, to the extent to which we are seeking to understand how people with severe trauma histories organize their current narrative of their past life, the chosen method is actually valid. PTSD as a disorder is often less about what actually happened in the past but more so about what is perceived to have happened and what the perceived implications are for the present (Dalgleish, 2004; Ehlers & Clark, 2000).

Another limitation relates to the samples used in the current analyses. The sample sizes for the two clinical groups were modest, as is often the case for hard-to-recruit clinical samples. However, there is nothing to indicate that the pattern and magnitude of the results relate to a lack of statistical power, and the sample sizes exceeded our a priori power estimates. The sexual-trauma-exposed sample was also all women. Finally, the trauma/no-MDD group contained some individuals with past experience of depression, although if anything, this would have been more likely to reduce between-group effects compared with the depression sample. Nevertheless, it would be important to replicate the current findings with both clinical groups in larger samples including individuals who have experienced different traumas, who are men, who have no lifetime history of depression, and if it is possible to find such individuals, who have severe trauma histories but no resulting psychopathology.

In summary, the present study used an established card-sorting task to examine the organization of autobiographical knowledge in a sample of sexual-trauma survivors with PTSD compared with a sample of individuals with chronic depression and with control participants. The sexual-trauma-exposed group with associated PTSD presented with a life structure significantly different to control participants and to participants with chronic depression, which supports proposals that the life narrative is organized differently in distressed survivors of severe trauma.

## Supplemental Material

Hitchcock_Supplemental_Material – Supplemental material for Fractured Pasts: The Structure of the Life Story in Sexual-Trauma Survivors With Posttraumatic Stress Disorder Click here for additional data file. Supplemental material, Hitchcock_Supplemental_Material for Fractured Pasts: The Structure of the Life Story in Sexual-Trauma Survivors With Posttraumatic Stress Disorder by Georgina Clifford, Caitlin Hitchcock and Tim Dalgleish in Clinical Psychological Science

## Appendix A

**Table A1.** Positive and Negative Words/Phrases Used in the Card Sorts

Positive		Negative	
Happy	Wisdom	Naïve	Insignificant
Satisfying	Accomplished	Incomplete	Insecure
Overjoyed	Important	Confused	Ashamed
Fulfilling	Feeling Together	Boring	Feeling Rejected
Successful	Exciting	Apathetic	Unfulfilling
Feeling Loved	Complete	Moody	Gloomy
Confident	Relaxed	Regretful	Feeling Unwanted
Creative	Feeling Courageous	Feeling Contaminated	Lonely
Feeling Needed	In Control	Stressful	Depressing
Passionate	Organised	Out of Control	Feeling Unloved
Feeling Nurtured	Stable	Unsuccessful	Hopeless
Joyful	Feeling Pure	Feeling Broken	Failure

## Appendix B

**Table B1.** Card Sort for a Participant With a More Integrated Card Sort (Φ = 0.53)

Mother & father	Grandmother	England	University	Sedentary life	First job	Second job
Exciting	Depressing	Incomplete	Depressing	Loved	Relaxed	Happy
Fulfilling	Unsuccessful	Sad	Unsuccessful	Wisdom	Fulfilling	Moody
Happy	Sad	Failure	Contaminated	Failure	Incomplete	Ashamed
Joyful	Lonely	Confused	Stressful	Sad	Insecure	Incomplete
Loved	Confused	Insecure	Naive	Passionate	Sad	Unfulfilling
Satisfying	Insecure	Successful	Loved	Successful	Moody	In control
Important	Naive	Broken	Out of control	Insecure	Ashamed	Confused
Relaxed	Stressful	Passionate	Moody	Incomplete	Accomplished	Joyful
	Rejected	Wisdom	Creative	Together	Unfulfilling	Complete
	Failure	Moody	Broken	In control	In control	Successful
	Unwanted	Nurtured	Wisdom	Accomplished	Stable	Hopeless
	Unfulfilling	Creative	Passionate	Stable	Stressful	Relaxed
	Contaminated		Sad	Fulfilling	Creative	Rejected
	Hopeless		Failure	Needed	Loved	Unsuccessful
	Incomplete		Confused	Satisfying	Wisdom	Out of control
				Exciting	Needed	Failure
				Important	Together	Exciting
				Happy	Happy	Passionate
				Relaxed	Courageous	Depressing
				Stressful	Satisfying	Wisdom
				Creative	Important	Insecure
					Failure	Sad
					Passionate	Confident
					Depressing	Needed
					Successful	Satisfying
					Confident	Loved
					Unsuccessful	
					Exciting	

Note: Chapter titles have been adapted slightly for the purpose of anonymity, though they remain faithful to the chapter content. Negative words are in bold.

**Table B2.** Card Sort for a Participant With a More Compartmentalized Sort (Φ = 0.90)

Infant school	Junior school	Senior school	University	Qualified	The break up	Sisterhood
Important	Exciting	In control	Exciting	Overjoyed	**Regretful**	Exciting
Fulfilling	Stable	Together	Nurtured	Exciting	**Failure**	Overjoyed
Joyful	Satisfying	Important	**Stressful**	Courageous	**Broken**	Accomplished
Happy	In control	Loved	**Confused**	Accomplished	**Rejected**	Stable
Exciting	Together	Passionate	Wisdom	**Out of control**	**Stressful**	Complete
Overjoyed	Important	Confident	Organised	Important	**Incomplete**	Satisfying
	Passionate	Pure	Important	Loved	**Confused**	**Confused**
	Confident	Successful	Loved	Passionate	**Insecure**	In control
	Pure	Fulfilling	Passionate	Successful	**Apathetic**	Organised
	Successful	Happy	Successful	Joyful	**Unloved**	Important
	Happy		Happy	Happy	**Unwanted**	Loved
	Creative		Creative	Creative	**Lonely**	Passionate
			Fulfilling	Fulfilling	**Sad**	Confident
						Pure
						Successful
						Joyful
						Happy
						Creative
						Fulfilling

Note: Chapter titles have been adapted slightly for the purpose of anonymity, though they remain faithful to the chapter content. Negative words are in bold.

## Transparency

*Action Editor:* Erin B. Tone

*Editor:* Scott O. Lilienfeld

*Author Contributions*

C. Hitchcock and T. Dalgleish should be considered joint senior authors. G. Clifford and T. Dalgleish designed the study. G. Clifford and C. Hitchcock completed data collection. All the authors contributed to data analysis and writing the manuscript. All the authors approved the final manuscript for submission.

## Notes

1. The original BDI was preferred here over updated versions for legacy reasons involving comparability of scores across time for the Cognition and Brain Sciences Unit Volunteer Panel.

2. Because the study samples differed overall on age, *F*(3, 102) = 7.46, *p* < .001, *d* = 0.51, 95% CI = [0.13, 0.89], and gender ratio, Freeman-Halton Fisher exact probability, *p* < .001 (see Table S3 in the Supplemental Material), we covaried age and gender for these and subsequent analyses comparing across the two data sets.
