# Supplementary material for: Fractured Pasts: The Structure of the Life Story in Sexual-Trauma Survivors With Posttraumatic Stress Disorder
Source: Clin Psychol Sci. 2020 Jun 18;8(4):723–38. doi: 10.1177/2167702620917984 (PMC7411540; doi:10.1177/2167702620917984)
Supplement: Hitchcock_Supplemental_Material – Supplemental material for Fractured Pasts: The Structure of the Life Story in Sexual-Trauma Survivors With Posttraumatic Stress Disorder [file Hitchcock_Supplemental_Material.pdf]

SUPPLEMENTAL MATERIAL

Fractured pasts: The structure of the life story in sexual trauma survivors with Posttraumatic Stress Disorder (PTSD)

Clifford, G.[1]

Hitchcock, C. [1]

Dalgleish, T. [1,2]

[1] Medical Research Council Cognition and Brain Sciences Unit, University of Cambridge, 15 Chaucer Road, Cambridge, CB2 7EF

[2]Cambridgeshire and Peterborough NHS Foundation Trust

All correspondence concerning this article should be addressed to Georgina Clifford, DClinPsy, or Tim Dalgleish, Medical Research Council Cognition and Brain Sciences Unit, 15 Chaucer Road, Cambridge, CB2 7EF, UK. E-mail: georgina.smith@mrc-cbu.cam.ac.uk or tim.dalgleish@mrc-cbu.cam.ac.uk

Supplemental Results

Sensitivity analysis comparing the PTSD/No MDD Group versus the No PTSD/No MDE Control Group on past life structure metrics

In these analyses we set aside the seven participants with a comorbid diagnosis of MDD from the original PTSD group and the three controls with a past Major Depressive Episode (MDE) from the original No PTSD Control Group and conducted sensitivity analyses on the remaining sample (n=20 per group; see Table S1 for past life structure metrics) in order to verify that these smaller groups still showed the same pattern of differences on our past life structure metrics. These sample sizes still fell within the acceptable estimations based on our *a priori* power calculations (see Participants section of the main manuscript).

Table S1: Means (standard deviation) of scores on the past life structure metrics for the PTSD/No MDD Group and the No PTSD/No MDD Control Group

	PTSD/No MDD Group (n=20) *Mean (SD)*	No PTSD/No MDE Control Group (n = 20) *Mean (SD)*
Prop. of Negative Cards	0.50 (0.16)	0.24 (0.14)
Negative Redundancy	0.27 (0.09)	0.24 (0.07)
Positive Redundancy	0.31 (0.12)	0.40 (0.15)
Compartmentalisation	0.76 (0.16)	0.54 (0.22)

Mirroring the data with the full sample (see main manuscript), these sensitivity analyses revealed a statistically significant multivariate difference in the past life structure components across groups, Wilk's Λ = 0.52, F (4, 35) = 8.13, P < .001; d = 0.90; 95% CIs

[0.22, 1.58]. The follow-up univariate ANOVAs revealed a significantly greater proportion of negative cards, F (1, 38) = 30.85, P < .001, d = 1.76; 95% CIs [0.99, 2.53], significantly greater compartmentalization, F (1, 38) = 12.67, P = .001, d = 1.13; 95% CIs [0.43, 1.83], and significantly reduced positive redundancy, F (1, 38) = 4.49, P = .04, d = 0.67; 95% CIs [0.002, 1.34], in the PTSD/No MDD group. There was no significant difference between groups for negative redundancy, F (1, 41) = 2.00, P = .17, d = 0.45; 95% CIs [-0.21, 1.11].

Sensitivity analysis comparing the MDD/No PTSD group against the No MDD/No PTSD Control group on past life structure metrics

Table S2: Means (standard deviation) of scores on the past life structure metrics for the MDD/No PTSD Group and the No MDD/No PTSD Control Group

	MDD/No PTSD) Group (*n*=30) *Mean (SD)*	No MDD/No PTSD Control Group (*n* = 36) *Mean (SD)*
Prop. of Negative Cards	0.61 (0.19)	0.28 (0.12)
Negative Redundancy	0.38 (0.19)	0.24 (0.12)
Positive Redundancy	0.26 (0.11)	0.46 (0.19)
Compartmentalisation	0.74 (0.18)	0.56 (0.23)

In these analyses we set aside participants with a diagnosis of current or past PTSD. Mirroring the original depression study findings (Dalgleish et al., 2011), there was a statistically significant multivariate difference in the past life structure components across groups, Wilk's Λ = 0.43, F (4, 61) = 20.23, P < .0001; d = 1.11; 95% CIs [0.57, 1.65]. The follow-up univariate ANOVAs revealed a significantly greater proportion of negative cards, F (1, 64) = 76.29, p <.0001, d = 2.16; 95% CIs [1.53, 2.79], significantly greater compartmentalization, F (1, 64) = 18.85, p < .0001, d = 1.07; 95% CIs [0.53, 1.61],

significantly greater negative redundancy, F (1, 64) = 18.85, P< .0001, d = 1.07; 95% CIs [0.53, 1.61], and significantly reduced positive redundancy, F (1, 64) = 28.19, p < .0001, d = 1.31; 95% CIs [0.76, 1.86], in the MDD (No PTSD) relative to the MDD study controls.

Table S3: Frequencies for gender and means (standard deviations) for age and depression symptom scores for the PTSD/No MDD group, the MDD/No PTSD Group, the No PTSD/No MDE control group and the No MDD/No PTSD control group

	PTSD/No MDD Group (*n*=20)	MDD/No PTSD Group (*n* = 30)	No PTSD/No MDE Control Group (*n* = 20)	No MDD/No PTSD Control Group (*n* = 36)
Gender *(female/male)*	20/0	20/10	20/0	28/8
Age in years	37.70 (13.65)	47.00 (11.80)	34.65 (15.91)	48.58 (10.33)
BDI Total Score	21.20^{ab} (9.36)	26.00^{cd} (11.68)	1.55^{ac} (1.84)	3.14^{bd} (4.02)

Note

^{abcd} Beck Depression Inventory (BDI) scores sharing the same superscript differ significantly from each other, Ps<.001.
